# Promptness in seeking treatment from Village Health workers for children under five years with malaria, diarrhoea and pneumonia in rural southwestern Uganda

**DOI:** 10.1186/s12936-023-04633-z

**Published:** 2023-06-27

**Authors:** Edgar Mulogo, Stephen Baguma, Moses Ntaro, Shem Bwambale, Michael Matte, Andrew Wesuta, David Ayebare, Fred Bagenda, Peter Kawungezi

**Affiliations:** 1 Department of Community Health, Mbarara University of Science and Technology, P.O. Box 1410, Mbarara, Uganda; 2 Bugoye Community Health Collaboration, P.O. Box 149, Kasese, Uganda; 3 Bugoye Health Centre, Kasese District Local Government, P.O. Box 149, Kasese, Uganda

**Keywords:** Promptness, Treatment, Childhood illness, Community health worker

## Abstract

**Background:**

Village Health Workers (VHWs) in Uganda provide treatment for the childhood illness of malaria, pneumonia, and diarrhoea through the integrated community case management (iCCM) strategy. Under the strategy children under five years receive treatment for these illnesses within 24 h of onset of illness. This study examined promptness in seeking treatment from VHWs by children under five years with malaria, pneumonia, and diarrhoea in rural southwestern Uganda.

**Methods:**

In August 2022, a database containing information from the VHWs patient registers over a 5-year study period was reviewed (2014–2018). A total of 18,430 child records drawn from 8 villages of Bugoye sub-county, Kasese district were included in the study. Promptness was defined a caregiver seeking treatment for a child from a VHW within 24 h of onset of illness.

**Results:**

Sixty-four percent (64%) of the children included in the study sought treatment promptly. Children with fever had the highest likelihood of seeking prompt treatment (aOR = 1.93, 95% CI 1.80–2.06, *p* < 0.001) as compared to those with diarrhoea (aOR = 1.43, 95% CI 1.32–1.52, p < 0.001) and pneumonia (aOR = 1.33, 95% CI 1.24–1.42, *p* < 0.001).

**Conclusion:**

The findings provide further evidence that VHWs play a critical role in the treatment of childhood illness in rural contexts. However, the proportion of children seeking prompt treatment remains below the target set at the inception of the iCCM strategy, in Uganda. There is a need to continually engage rural communities to promote modification of health-seeking behaviour, particularly for children with danger signs. Evidence to inform the design of services and behaviour change communication, can be provided through undertaking qualitative studies to understand the underlying reasons for decisions about care-seeking in rural settings. Co-design with communities in these settings may increase the acceptability of these services.

## Background

In 2019 an estimated 5.2 million children under 5 years died mostly from preventable and treatable causes. These include; malaria, diarrhoea and pneumonia that can be prevented or treated with access to quality care by a trained health provider when needed, among other interventions [1]. The World Health Organization (WHO) estimates that a total of 33 million cases of malaria, diarrhoea and pneumonia go untreated every year. Early or timely access to medical care is one of the core approach in elimination of these illnesses in sub-Saharan Africa [2]*.* In order to decrease severity of childhood illnesses and its subsequent death, there is need for improving access to health workers [3] and appropriate health care-seeking behaviour of caregivers [4].

Diarrhoea, malaria, and pneumonia are the leading causes of mortality in children under-5 years of age in Africa [5, 6]. Sub-Saharan Africa and South Asia have registered the lowest reductions in mortality rates as more than 7 million children under age five (“U5s”) die every year of pneumonia, malaria and diarrhoea. In 2012, these regions contributed about 82% of the global under-five mortality. The two leading death causes of under-five children is pneumonia, accounting for 18% followed by diarrhoeal accounting for 15% of the total under-five mortality. For only malaria, over 90% globally occur in sub-Saharan Africa [7]. The region has age-long prevalence of under-5 mortalities despite increasing health intervention [8].

Evidence shows that most malaria related deaths in malaria affected countries occur at home without receiving appropriate or timely medical care, and when care is sought, it is often delayed [9]. Delay in seeking healthcare has been shown in several regions to play an important role in under-five morbidity and mortality [10]. A number of studies conducted in developing countries have also shown that delay in seeking appropriate care or not seeking any care causes a large number of child deaths [11]. Late care-seeking contributed to high mortality from acute respiratory infections in Uganda and from malaria in Tanzania. Even when drugs are free at community level caregivers do not seek treatment in time [12]. Other caregivers on contrary first use herbs and after this treatment fails they take their child to health workers when the situation has worsened [13]. In sub-Saharan Africa, responses to seek care outside home are often delayed, and when a response is made is at village drug shops characterized by inadequate trained workers [14, 15].

In Uganda alone, about 60% of parents with febrile children first seek care in the private sector [16, 17]. In most rural areas caregivers hardly recognize signs of childhood illness for timely care-seeking [18]. Delay in seeking health care for a sick infant has been attributed to several factors for example combining home remedies with conventional treatments, inability to identify life-threatening illnesses and lack of knowledge. These challenges exist against a background of undiagnosed serious life threatening illness such as diarrhoea, and malaria [19].

In recent years, there has been a resurgence of interest by the international community in the use of VHWs as a way to bridge the gap in reducing child mortality through introducing iCCM as a way of timely handling of cases at village level. In iCCM model, trained VHWs are typically equipped to timely assess and treat the leading infectious causes of child mortality under 5 years [2, 20]. VHWs are trained, equipped with medicine and diagnostic gadgets to use at the community level. They also identify and refer children with severe disease to health facilities [21]. Government of Uganda with support from partners introduced iCCM to offer timely curative treatments for malaria, diarrhoea and pneumonia at community level. This was aimed at timely diagnosis and treatment to those who can hardly access health facilities [22]. However, the success of iCCM solidly depends on caregivers who should timely be able to recognize symptoms, seek health care from CHWs and adhere to treatment or referral within 24 h.

Despite progress made in reducing child mortality in Uganda, some caregivers’ choice for seeking and utilization of health care provided by community health workers is associated with severity of illness [23]. iCCM has the potential to increase access to prompt care, thereby decreasing morbidity and mortality [24]. A key objective of the iCCM programme in Uganda was to increase to at least 80% the proportion of children under-five years receiving appropriate treatment for malaria, pneumonia and diarrhoea within 24 h of onset of illness [25].

However, there is limited information on the promptness in seeking health treatment from VHWs, for children under five years with malaria, pneumonia and diarrhoea. This study, therefore, examined the promptness in seeking treatment for children under five years in a rural community.

## Methods

### Design and setting

A retrospective data review of records of VHWs in 8 villages of Bugoye sub-county was conducted in 2022. The review covered a period of five years (2014–2018). Bugoye Sub-county, Uganda, is a rural, mountainous area in Kasese District (on the western border of Uganda), VHWs from the national programme have provided iCCM care since 2013, with financial and operational support from a long-standing collaboration with Mbarara University of Science and Technology (Mbarara, Uganda) and the Massachusetts General Hospital (Boston, Massachusetts, USA) [26].

The Bugoye VHWs were enlisted, trained and supplied to provide iCCM services in 22 villages of the sub-county, surround Bugoye Health Centre level III. Bugoye sub-county is home just over 45,000 people with an average household size of 5.6 people, with malaria and respiratory infections being the most predominant childhood illnesses. The VHW team is elected by its village community, with four to five VHWs derived from each of the villages. They receive comprehensive training to appropriately diagnose, treat and/or refer children under five years old with malaria, pneumonia or diarrhoea. Initially, VHWs participate in a basic 3-day training standardized by the Ugandan Ministry of Health (MoH) to review VHW responsibilities. This was followed with a 6-day training on implementing iCCM services [27].

The VHWs use the iCCM protocol, called the “Sick Child Job Aid”, to determine the proper care for each patient. They are equipped with rapid diagnostic tests (RDT) for malaria diagnosis in patients presenting with subjective fever; pneumonia is diagnosed based on age-based respiratory rate cut-offs, and diarrhoea is diagnosed by clinical history. For patients with “danger signs” (evidence of severe illness), VHWs provide initial assessment and referral or accompaniment to a health facility, as well as pre-referral treatment for some conditions. The five general danger signs include; vomiting everything, chest in-drawing, convulsions, not able to breast feed or drink, and very sleepy/unconscious/difficulty to wake [28]. VHWs use a “Sick Patient Register” to record each patient they assess. These Sick Patient Registers are submitted monthly and tallied to create a “Monthly Report” for the overall programme. The filed registers and monthly reports provided the data sources for this research.

### Data collection

The filed registers provide the following information on the following; basic information about the patient (age, sex and presenting complaints), clinical assessment data (presence of danger signs, respiratory rate and malaria RDT result), actions taken (medications administered and/or referral to the health facility), as well as whether the patient was seen by the VHW within 24 h of onset of symptoms. An electronic database was created using de-identified data for all encounters from April 2014 to December 2018.

### Variables and analysis

The study variables were derived from the information provided in the Sick Patient Registers. These variables include; sex, age, RDT result, whether the child had; diarrhoea, fast breathing, fever, danger sign, or was treated within 24 h. “Promptness” was defined as children seeking treatment from a VHW within 24 h of onset of illness (malaria, pneumonia, diarrhoea).

The data analysis was conducted using STATA version 14 software [29]. Two-sided chi-square tests for association were computed to detect differences between proportions of categorical variables such as sex, presenting compliant, presence of danger signs, referral, and whether patient visited the VHW within 24 h of onset of illness. The means of continuous variables such as age were compared using t-tests. A sliding scale of p-values was used as a basis to interpret the strength of association.

Logistic regression models were run to investigate the relationship between the outcome variable (promptness) and other variables. The model building strategy was not only limited to significant variables from the bivariate analysis, but also included independent variables that were considered to have social plausibility to promptness in seeking care [30].

## Results

### Background characteristics of the children

A total of 18,430 child encounters were analysed. Of these 11,724 (64%) visited the CHWs within 24 h. The background characteristics of the children are shown in Table 1. The majority of the children that had fever, diarrhoea and pneumonia visited the VHWs within 24 h of onset of illness (p < 0.05). Only 37% of the children with danger signs visited the VHWs within 24 h of onset of illness (p < 0.001).

**Table 1:** Background characteristics of children

Characteristic	All (N = 18,430)	Visited VHW within 24 h		p-value
	n (%)	No (6706) n (%)	Yes (11,724) n (%)	
Mean age in months (SD)	28.1 (± 17.1)	27.6 (± 17.6)	28.3 (± 16.9)	0.004^**¥^
Sex (n = 18,297)				0.428
Female	9,105 (50)	3,266 (36)	5,839 (64)	
Male	9,192 (50)	3,349 (36)	5,843 (64)	
Fever (n = 18,430)				< 0.001^**^
No	7,618 (41)	3,314 (44)	4,304 (56)	
Yes	10,812 (81)	3,392 (31)	7,420 (69)	
Diarrhoea (n = 18,430)				< 0.001^**^
No	13,260 (72)	4,978 (38)	8,282 (62)	
Yes	5,170 (28)	1,728 (33)	7,420 (67)	
Pneumonia (n = 18,430)				0.026^*^
No	10,091 (55)	3744 (37)	6347 (63)	
Yes	8339 (45)	2962 (36)	5377 (64)	
Child had danger sign (n = 18,430)				< 0.001^***^
No	18,201(99)	6561(36)	11,640 (99)	
Yes	229 (1)	145 (63)	84 (37)	

*SD* standard deviation, ¥  *t* test

*significant at p < 0.05, **significant at p < 0.01 and ***significant at p < 0.001

### Factors associated with promptness in seeking care from VHWs

The factors associated with promptness in seeking care from VHWs are shown in Table 2.

**Table 2:** Association between patient characteristics and promptness in seeking care

Characteristics	Visit CHWs within 24 h		OR (95% CI)	p-value	aOR (95% CI)	p-value
	No	Yes				
Age						
< 12 months	1415	2060	1 1.25 (1.16–1.35)	< 0.001^***^		
≥ 12 months	5291	9664			1.16 (1.07 – 1.25)	< 0.001***
Sex						
Male	3349	5843	1 1.02 (0.96–1.08)	0.42		
Female	3266	5839			1.01 (0.95 – 1.07)	0.629
Fever						
No	3314	4304	1 1.68 (1.58–1.78)	< 0.001^***^		
Yes	3392	7420			1.93 (1.80 – 2.06)	< 0.001***
Diarrhoea						
No	4978	8282	1 1.19 (1.11–1.28)	< 0.001^***^		
Yes	1728	3442			1.43 (1.32 – 1.54)	< 0.001***
Pneumonia						
No	3744	6347	1 1.07 (1.00–1.13)	0.02^**^		
Yes	2962	5377			1.33 (1.24 – 1.42)	< 0.001***
Child had danger sign						
No	6561	11,640	1 0.32 (0.24–0.42)	< 0.001^***^		
Yes	143	84			0.51 (0.38 – 0.69)	< 0.001***
Child referred						
No	6124	11,212	1 0.48 (0.42–0.54)	< 0.001^***^		
Yes	576	510			0.59 (0.52 – 0.68)	< 0.001***

The patient characteristics independently associated with promptness in seeking care from a VHW are; fever (aOR = 1.93, 95% CI 1.80–2.06, *p* < 0.001), diarrhoea (aOR = 1.43, 95% CI 1.32–1.54, *p* < 0.001), pneumonia (aOR = 1.33, 95% CI 1.24–1.42, p < 0.001), child had danger sign (aOR = 0.51, 95% CI 0.38–0.69, p < 0.001), and child referred (aOR = 1.59, 95% CI 0.52–0.68, p < 0.001)

**significant at p < 0.01 and ***significant at p < 0.001

## Discussion

The study findings show that the majority of children sought treatment from the VHWs within 24 h of illness. This is consistent with findings elsewhere that have reported promptness in seeking treatment from VHWs [28, 31]. Prompt seeking of treatment from the VHWs has been associated with short distance to reach the VHW, where the mother was the decision maker in the household, high trust and satisfaction with the services provided [28, 32]. The proportions of children with fever, diarrhoea and pneumonia who sought care from the VHWs within 24 h of onset of symptoms was similar, but much lower than the national average at the time [25]. This may be attributed to the rugged mountainous terrain in which the communities dwell in comparison to other areas of the country, that may result in delays by some caregivers in seeking care for the children.

Children with fever sought treatment more promptly in comparison to those with diarrhoea and pneumonia. However studies elsewhere have reported that children with fast breathing seek treatment more promptly than those with either fever or diarrhoea [28, 31]. This study noted that children with danger signs were less likely to seek treatment promptly. It is assumed that children with danger signs, which often reflects more severe illness would be expected to seek treatment promptly. However, it has been reported that caregivers often do not recognize symptoms and danger signs of severely ill children, which may lead to delays in seeking treatment [33, 34]. On the other hand, a number of children with danger signs bypass the VHWs and go directly to the health facilities which is the appropriate action.

## Limitations

Although the study data is from an iCCM programme in one sub county the context and the providers are typical of other rural setting in Uganda. The study findings are therefore comparable across similar settings in the country. The study data is also cross-sectional in nature therefore a temporal relationship between the independent variables and outcome cannot be elucidated. The direction of causality can therefore only be regarded as suggestive.

## Conclusions

The findings provide further evidence that VHWs play a critical role in the treatment of childhood illness in rural contexts. However, the proportion of children seeking prompt treatment remains below the target set at the inception of the iCCM strategy, in Uganda. There is a need to continually engage rural communities to promote modification of health-seeking behaviour, particularly for children with danger signs. Evidence to inform the design of services and behaviour change communication, can be provided through undertaking qualitative studies to understand the underlying reasons for decisions about care-seeking in rural settings. Co-design with communities in these settings may increase the acceptability of these services.

## Data Availability

All data supporting our findings are contained in the paper. There are no restrictions to data sources, however, details of the full data may be accessed through Prof. Edgar Mugema Mulogo (corresponding author), Department of Community Health, Mbarara University of Science and Technology, PO Box 1410, Mbarara, Uganda.
